# Non-thermal obliteration of critically ranked carbapenem-resistant *Acinetobacter baumannii* and its resistance gene in a batch atmospheric plasma reactor

**DOI:** 10.1007/s11356-024-34475-4

**Published:** 2024-07-31

**Authors:** Thabang B. M. Mosaka, John O. Unuofin, Michael O. Daramola, Chedly Tizaoui, Samuel A. Iwarere

**Affiliations:** 1 Sustainable Energy and Environment Research Group, Department of Chemical Engineering, Faculty of Engineering, Built Environment and Information Technology, University of Pretoria, Hatfield, 0002 Pretoria South Africa; 2 Water and Resources Recovery Research Lab, Department of Chemical Engineering, Faculty of Science and Engineering, Swansea University, Swansea, SA1 8EN UK

**Keywords:** *Acinetobacter baumannii*, Carbapenem-resistant gene, Cold atmospheric plasma, Disinfection

## Abstract

Wastewater treatment plants (WWTPs) have been implicated as direct key reservoir of both antibiotic-resistant bacteria (ARB) and antibiotic-resistant genes (ARGs) associated with human infection, as high concentrations of ARBs and ARGs have been detected in recycled hospital wastewater. Among the ARBs, the carbapenem-resistant *Acinetobacter baumannii* has been ranked as priority 1 (critical) pathogen by the World Health Organization (WHO), due to its overwhelming burden on public health. Therefore, this study is aimed at investigating non-thermal plasma (NTP) technology as an alternative disinfection step to inactivate this bacterium and its ARGs. Culture-based method and PCR were employed in confirming the carbapenem resistance gene *bla*_NDM-1_ in *A. baumannii* (BAA 1605). Suspension of carbapenem-resistant *A. baumannii* (24 h culture) was prepared from the confirmed isolate and subjected to plasma treatment at varying time intervals (3 min, 6 min, 9 min, 12 min, and 15 min) in triplicates. The plasma-treated samples were evaluated for re-growth and the presence of the resistance gene. The treatment resulted in a 1.13 log reduction after 3 min and the highest log reduction of ≥ 8 after 15 min, and the results also showed that NTP was able to inactivate the *bla*_NDM-1_ gene. The log reduction and gel image results suggest that plasma disinfection has a great potential to be an efficient tertiary treatment step for WWTPs.

## Introduction

Today, clinical isolates of *Acinetobacter baumannii*, resistant to carbapenems (last resort antibiotics), are being reported globally and have earned the term “red alert” human pathogen, among the medical fraternity (Howard et al. 2012; Dekic et al. 2019). The World Health Organization (WHO) even categorized *A. baumannii*, carbapenem-resistant as critical priority 1 class (World Health Organisation 2024; Soni et al. 2022). *A. baumannii* is a gram-negative bacterium, that is, non-motile, pleomorphic, and strictly aerobic (Howard et al. 2012; Viehman et al. 2014; Valencia-Martín et al. 2019; Raut et al. 2020; Shi et al. 2020). It is commonly associated with aquatic environments (Howard et al. 2012), but it is also notorious for surviving desiccation and surviving for prolonged periods on all kinds of surfaces (dry and wet) (Fishbain and Peleg 2010; Viehman et al. 2014; Valencia-Martín et al. 2019; Raut et al. 2020). This key feature facilitates its dissemination within the health care setting and it often leads to outbreaks (Fishbain and Peleg 2010; Viehman et al. 2014), which are rampant among immunocompromised individuals, especially patients and convalescing persons who have been in the hospital for a long time (> 90 days)*.* Causing life-threatening infections such as respiratory tract infection, bacteremia, meningitis, urinary tract infections, and wound infection have limited options for treatment (Fishbain and Peleg 2010; Howard et al. 2012; Valencia-Martín et al. 2019; Raut et al. 2020; Shi et al. 2020). These clinical manifestations often result in patients being admitted to the intensive care unit, having surgical procedures done on them, and being hospitalized for longer periods (Fishbain and Peleg 2010). In critical cases, it eventually leads to the demise of suffering patients (Raut et al. 2020). *A. baumannii* has developed resistance to commonly used antibiotics during the last 30 years (Dekic et al. 2019), accentuating its status amongst the most common and serious multi-drug resistant (MDR) bacteria, ESKAPE (*Enterococcus faecium*, *Staphylococcus aureus*, *Klebsiella pneumoniae*, *A. baumannii*, *Pseudomonas aeruginosa*, and *Enterobacter* spp.). Its intrinsic resistance mechanisms, together with the acquisition of foreign determinants, enable it to switch its genomic structure, quickly capturing resistance markers under antibacterial pressure (Howard et al. 2012).

Over the last decade, viable *A. baumannii* of clinical significance has been reported in natural environments outside of hospital settings, being recovered in rivers, wastewater treatment plants, untreated hospital wastewaters, and urban wastewaters. There is, however, very little information about the environmental factors that influence the survival of *A. baumannii* in waters from which it was found (Dekic et al. 2019). Most studies have investigated and observed the presence of carbapenem-resistant *A. baumannii* (CRAB) after secondary treatment in WWTPs (Pulami et al. 2023). One of the few studies that have investigated the effectiveness of disinfection have reported CRAB and the NDM-1 gene in the effluent of the WWTP after chlorine disinfection (Hrenović et al. 2016). This is because chlorination tends to have a selective effect on antibiotic-resistant genes (ARGs), decreasing abundance of genes (gene copies per mL of sample) while increasing the prevalence of the gene (gene copies per total bacteria) (Manaia et al. 2018; Chen et al. 2020b). The ARGs are eventually transferred and adapted into new bacteria, leading to the inception and genetic transformation across bacteria and the development of antibiotic resistance (Yuan et al. 2015; Sarangapani et al. 2019; Chen et al. 2020b; Jin et al. 2020).

The inability of WWTPs to inactivate antibiotic-resistant bacteria (ARBs) and ARGs is not limited to chlorination; studies have proven that when either chlorination or UV irradiation or ozone oxidation is applied in WWTPs, they might destroy the bacteria by disintegrating its DNA or cellular structure. However, ARGs may still persist for a long time in the cell debris and in the environment, eventually resulting in bacteria developing antibiotic resistance (Yuan et al. 2015; Sarangapani et al. 2019; Chen et al. 2020b; Jin et al. 2020). This recycled drinking water becomes a direct key reservoir of ARBs and ARGs associated with human infection (Ekwanzala et al. 2018), making wastewater both a resource and a problem (Unuofin 2020). One of the emerging and promising technology in the treatment of water and wastewater is the use of non-thermal plasma discharges. Non-thermal plasma has found great applications in the last two decades from fields such as medicine (plasma medicine), agriculture (plasma agriculture), semi-conductor, plasma etching, surface modification of different materials, and synthesis of nanoparticles amongst many others. Although its application in the inactivation of certain gram-negative and gram-positive bacteria has been explored in the last decades (Gwanzura et al. (2021)), there are very few studies that have explored cold plasma with regard to the deactivation of antibiotic-resistant bacteria and their genes. Therefore, this study is aimed at using non-thermal plasma (NTP), a type of advanced oxidation process (AOP), as an alternative tool for both water treatment and wastewater reclamation and reuse, as it is able to produce reactive oxygen species (ROS) like the indiscriminate hydroxyl radical •OH, breaking down organic matter while inactivating ARBs and ARGs (Foster 2017; Chen et al. 2020c; Umar 2022). In particular, this study appraises the effectiveness of NTP on treating a saline suspension of planktonic typed strain, *Acinetobacter baumannii* ATCC BAA 1605, based on the concentration of its by-products, pH, and conductivity.

## Materials and methods

### Non-thermal plasma reactor

**Fig. 1:**
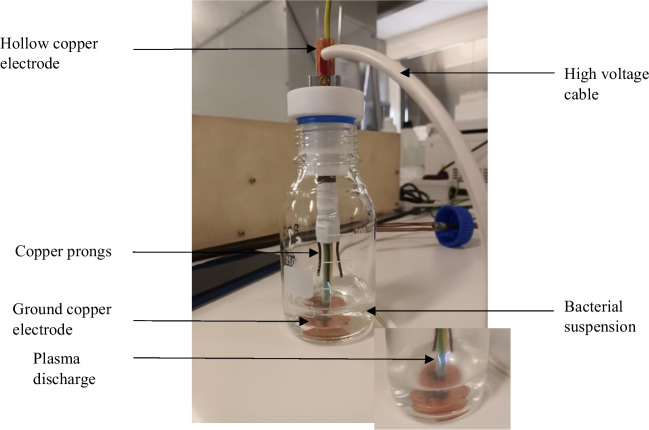
The non-thermal plasma batch reactor setup used for treatment

A 250 mL capacity Schott bottle fitted with a designed air-tight machined polytetrafluoroethylene (PTFE) was set up as a batch reactor for the treatment of samples (Fig. 1). Other components associated with the experimental setup are copper electrodes and high-voltage cable. The PTFE, which sealed the rector, was machined to create an orifice, which was fitted with a hollow copper electrode with an outer diameter of 12.7 mm. One end of the hollow copper electrode had four copper prongs (30 mm × 3 mm) welded on to it. The hollow electrode was connected to a high voltage end of a high voltage direct current power supply designed and manufactured by Jeenel Technology Services (Pty) Ltd in South Africa. The power supply has a maximum capacity of 40 kV and 15 mA. The ground electrode was a flat copper disk and had a gap of 50 mm between it and the copper electrode (prongs). The gap between the surface of the solution and the prongs was 15 mm. To initiate the plasma discharge, the voltage was set at 23 kV, but it went down to 10 kV after the initialization of the discharge, and current of 0.7 mA, sustaining a 7 W discharge power throughout the treatment. The insert at the bottom shows the discharge generated which is a streamer in nature. The white masking tape was wrapped around the hollow copper electrode to prevent arc formation at the neck of the reactor.

### Organism and reagents

The ATCC BAA 1605 type strain of *A. baumannii* adopted for this study was purchased from Laboratory Specialties PTY LTD Trading as Thermo Fisher Scientific in Randburg, South Africa. It was originally isolated in a Canadian hospital from the sputum of a military returnee from Afghanistan and was characterized as multi-drug resistant (ATCC, USA). Imipenem antibiotic, Luria Bertani (LB) broth, and LB agar were purchased from Sigma Merck in South Africa. Nucleomag DNA/RNA water kit was procured from Separations in Randburg, South Africa. Primers were delivered by Inqaba in Pretoria, South Africa. All reagents were of analytical grade.

### Antibiotic screening and non-thermal plasma

#### Antibiotic screening

The study confirmed the resistance of *A*. *baumannii* using culture methods (Rashmei et al. 2016). The bacteria were cultured in Luria Bertani (LB) broth and incubated under orbital conditions (160 rpm) at 37 °C for 24 h. Serial passaging on LB agar plates supplemented with increasing concentrations of imipenem (2 µg/mL, 4 µg/mL, 8 µg/mL, and 16 µg/mL) confirmed resistance (Ebomah and Okoh 2020; Reinke et al. 2020), particularly when growth occurred at 16 µg/mL imipenem. Isolates thriving under these conditions were identified as the standard carbapenem-resistant bacterial strain (CRBS) for subsequent inactivation experiments in the study.

#### Colony count and non-thermal plasma treatment of *Acinetobacter baumannii*

The standard *A. baumannii* (1 mL) was inoculated into nutrient broth (2 L) and prepared according to manufacturer’s instructions. The standard was incubated at 37 °C for 24 h (aerobically at 160 rpm). After incubation, the solution was centrifuged at 4500 rpm for 5 min to retrieve the bacteria pellets. The pellets were then washed twice with physiological saline solution and centrifuged at 4500 rpm for 5 min after each wash. Thereafter, the pellets were resuspended in 2 L physiological saline solution. The solution (1 mL) was plated on LB agar plates containing 16 µg/mL imipenem antibiotic and incubated at 37 °C for 48 h. The colonies were counted which gave an average plate count of 5.5 × 10^9^ CFU/mL before plasma treatment. The volume of treated water samples was 50 mL per treatment time. The plasma treatment was done for different durations in a range of 3, 6, 9, 12, and 15 min (Rashmei et al. 2016) in triplicate. After plasma treatment, the samples were plated on LB agar plates containing 16 µg/mL imipenem antibiotic, and incubated at 37 °C for 48 h. After incubation, the colony-forming units (per mL) were determined and used for log reduction calculations (Reinke et al. 2020). Copper is known to have antimicrobial properties (Benhalima et al. 2019; Ortega-Nieto et al. 2023); therefore, in order to check if it assisted the plasma discharge, the bacterial suspensions were exposed to a copper electrode, without electric discharge for 15 min.

### Physiochemical and structural characterization

The Black Comet C-25 Spectrometer (StellarNet, Inc) was used to investigate the discharge characteristics and the formation of the chemical species. The ${\text{H}}_{2}{\text{O}}_{2}$ and nitrite/nitrate ions gave absorbances at < 350 nm, but they are usually not obvious on the optical emission spectra. In order to obtain more accurate results (Zhang et al. 2021), the Lovibond® SpectroDirect single-beam spectrophotometer for water testing (Tintometer Group, Germany) was used to determine the concentration of (${\text{H}}_{2}{\text{O}}_{2}$), (${\text{NO}}_{2}^{-}$), and (${\text{NO}}_{3}^{-}$) after treating the bacterial suspensions with CAP for 3, 9, and 15 min. Different reagents were reacted with constant volume (10 mL) of the bacterial suspensions, for measurements of ${\text{H}}_{2}{\text{O}}_{2}$ and ${\text{NO}}_{2}^{-}$, while only 0.5 mL of the bacterial suspensions was used for measurements of ${\text{NO}}_{3}^{-}$. For ${\text{H}}_{2}{\text{O}}_{2}$ measurements, titanium tetrachloride reagent was used, whereas for ${\text{NO}}_{2}^{-}$ and ${\text{NO}}_{3}^{-}$ measurements, N-(1-Naphthyl)-ethylenediamine and 2,6-dimethylphenole reagents were used, respectively. Moreover, the reaction time and spectrophotometric reading related to the observed species were carried out according to the manufacturer’s manual (GmbH 2021). The PL-700AL pH meter was used to determine the pH, and the WTW Cond 3310 was used to determine the conductivity of the bacterial suspension before the plasma treatment and after 3, 6, 9, 12, and 15 min of the plasma treatment.

#### Scanning electron microscopic (SEM) analysis

The bacterial suspensions (before NTP and 15 min after respective treatments) were centrifuged for 5 min at 4500 rpm, the supernatant was discarded, and the pelleted cells were retrieved. Phosphate washing buffer was used to wash serum/media away for 15 min, the buffer was then removed by centrifuging, and the cells were retrieved; afterward, 2.5% of glutaraldehyde/formaldehyde solution was added and fixed for 1–24 h. The fixative solution was removed, the pellets were washed with phosphate washing buffer 3 times (15 min for each wash), and the buffer was removed. Then, 1% of osmium tetroxide (OsO_4_) solution and post-fixing was done for 1 h. OsO_4_ fixative solution was removed in the fume cupboard, and the first buffer wash was added in the fume cupboard. Washing was done with phosphate washing buffer 3 times (15 min for each wash), and the buffer was removed. A graded series of ethanol (30%, 50%, 70%, 90%, and 3X 100%) for 15 min each was used to dehydrated the pellets. The pellets were left in the last 100% ethanol for 30 min. A 50:50 mixture of HMDS and 100% ethanol was added and left for 1 h (sample was covered). The ethanol: HMDS mixture was removed, and HMDS was added and left for 1 h (sample was covered). HMDS was removed and fresh one was added; the container was left open for samples to dry. The samples were mounted onto aluminum stubs and coated with carbon and then examined in the SEM (Zeiss Gemini Ultra Plus FEG-SEM (field emission gun – scanning electron microscope) with BS, energy dispersive spectroscopy (EDS), and electron backscatter diffraction (EBSD) detectors).

### Molecular analysis

DNA was extracted from the bacterial suspension before and after NTP treatment using the kit according to Nucleomag’s instructions. The extracted DNA was used as template DNA for the PCR assay to confirm the presence of *bla*_NDM-1_ gene in *A. baumannii*. The primer used in this study can be found in Table 1.

**Table 1:** Primer used in this study

Name	Forward primer (5′–3′)	Reverse primer (5′–3′)	Size (bp)	Reference
*bla*_NDM-1_	GGTGCATGCCCGGTGAAATC	ATGCTGGCCTTGGGGAACGS	660	(Anand et al. 2015)

The PCR mixture contained 5 µL of PCR master mix with chosen 0.5 µL of forward primer, 0.5 µL of reverse primer, 2.5 µL of template DNA, and 1.5 µL of milli-q water to make up a reaction volume of 10 µL. The PCR conditions for *bla*_NDM-1_ were initial denaturation at 95 °C for 3 min, followed by 30 cycles for 1 min at 95 °C, annealing at 55 °C for 1 min, and extension at 72 °C for 1 min 30 s, with a final extension at 72 °C for 10 min. The BIO RAD T100 Thermal cycler was used. The PCR products were stained with ethidium bromide (Anand et al. 2015; Odjadjare and Olaniran 2015) and observed using electrophoresis in 1% agarose gel (Querci et al. 2020). According to Lee et al. (2012), the most efficient method of separating DNA fragments is agarose gel electrophoresis and the addition of ethidium bromide allows fluorescence of DNA under UV light. The BIO RAD PowerPac basic with Mini Sub Cell GT was used for electrophoresis. The presence or absence of the genes on the gel images gave an indication of the ability of plasma treatment to inactivate resistance genes (Anand et al. 2015).

## Results and discussion

### Physicochemical properties of plasma discharge

**Fig. 2:**
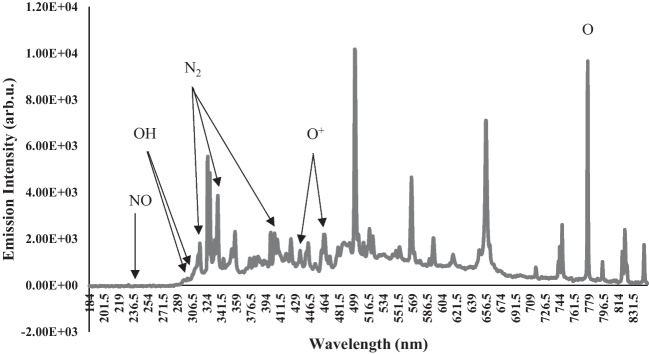
Optical emission spectra from hydroxyl radical OH species in non-thermal plasma during treatment of *A. baumannii*

NTP produces reactive nitrogen species (RNS) such as nitrites (NO_2_^−^) and nitrate (NO_3_^−^) (Sanito et al. 2022; Sreedevi and Suresh 2023; Zhang et al. 2023) and reactive oxygen species (ROS) such hydroxyl (OH) and hydrogen peroxide (H_2_O_2_) (Sreedevi and Suresh 2023; Sanito et al. 2022) in both the adjoining gaseous and liquid mediums (Sreedevi and Suresh 2023). The ROS and RNS play an important role in the inactivation of bacteria (Domonkos et al. 2021; Das et al. 2022). Figure 2 indicates that the NTP reactor generated a streamer discharge that mainly consisted of ROS and RNS, such as NO line (239.5 nm), O^+^ lines (435.5 nm and 464.5 nm), OH lines (307.5 nm, 309 nm), N_2_ lines (316 nm, 337 nm, and 404 nm), N^+^ lines (344.5 nm and 394.5 nm), and O line (777.5 nm). The oxygen (O_2_) and nitrogen (N_2_) present in the atmospheric air individually undergo electron impact ionization reactions, resulting in electrons and positive ions (O_2_^+^ and N_2_^+^) that separate and eventually enable streamer propagation which resulted in the formation of NO, O^+^, N^+^, and O in the gas phase (Nijdam et al. 2020). In wet air or in liquids, the •OH, H_2_O_2_, and ozone (O_3_) are formed. The nitrogen oxides (NO) dissolve in water forming nitrite ions and nitrate ions (Zhang et al. 2023). Among ROS, •OH has the highest oxidation potential, the most reactive, and is considered to play an important role in NTP bacterial treatment (Beber de Souza et al. 2015; Magureanu et al. 2021; Zhang et al. 2023). The strong oxidation potential (2.8 V) of the •OH is higher than the conventional disinfectants, chlorine (1.36 V), and ozone (2.07 V), and it can damage DNA (Foster 2017; Sharma et al. 2019; Rekhate and Srivastava 2020; Magureanu et al. 2021; Azuma et al. 2022). The •OH radical has diverse impact on normal protein structure which is one of the primary targets in bacteria during disinfection, including oxidation of amino acids and modification of sulfur groups, causing irreversible damage to cells and inactivation of ARB and ARGs (Chen et al. 2020c; Sreedevi and Suresh 2023).

**Fig. 3:**
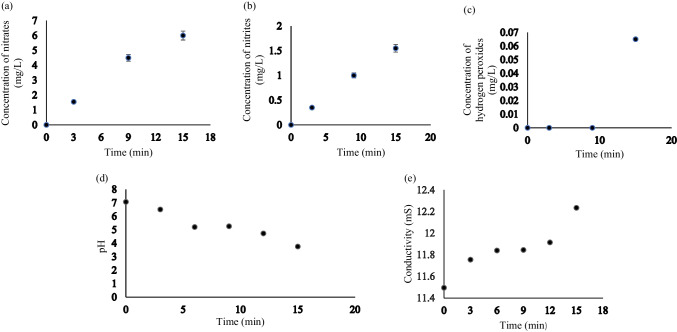
**a–e** Concentration of **a** nitrates, **b** nitrites, and **c** hydrogen peroxide during non-thermal plasma treatment of *A. baumannii*. **d** pH and **e** conductivity readings of *A. baumannii* bacterial suspension during non-thermal plasma treatment

Treatment time resulted in an increase of both concentrations of nitrates (${\text{NO}}_{3}^{-}$) and nitrites (${\text{NO}}_{2}^{-}$). The highest concentration for both species was observed at 15 min, the concentrations were 6 ± 0.3 mg/L and 1.55 ± 0.078 mg/L for ${\text{NO}}_{3}^{-}$ (Fig. 3a) and ${\text{NO}}_{2}^{-}$ (Fig. 3b), respectively. This correlated with a study that showed an increase in the concentration of nitrates and nitrites with time, although they attained a concentration of 41.41 mg/L and 5.27 mg/L of ${\text{NO}}_{3}^{-}$ and ${\text{NO}}_{2}^{-}$, respectively, after the same treatment time. Their reactor configuration was a dielectric barrier discharge with deionized water (Pandey et al. 2023). Nitrites tend to be oxidized to nitrates (Picetti et al 2022), which explains why nitrates were higher than nitrites in this study.

A concentration of 0.065 mg/L hydrogen peroxide (${\text{H}}_{2}{\text{O}}_{2}$) was only observed after 15 min of plasma treatment (Fig. 3c). A study resulted in nil production of ${\text{H}}_{2}{\text{O}}_{2}$ after plasma treatment (Pandey et al. 2023), while in another study, ${\text{H}}_{2}{\text{O}}_{2}$ was observed immediately after treatment, but the concentration decreased with the time of incubation (Sreedevi and Suresh 2023). ${\text{H}}_{2}{\text{O}}_{2}$ usually increases quadratically or linearly with plasma treatment time, but the cells in the medium uptake it with incubation time (Pandey et al. 2023; Sreedevi and Suresh 2023).

The (${\text{H}}_{2}{\text{O}}_{2}$), (${\text{NO}}_{2}^{-}$), and (${\text{NO}}_{3}^{-}$) have a relatively long lifetime and can react to secondary products post-discharge. The post-discharge reactions between the by-products occurring in plasma-activated water (PAW) can result in the generation of peroxynitrous (HNO_3_)/peroxynitrite (ONOO^−^) acid, which significantly participates in the antibacterial activity of PAW. The long-lived reactive species result (Rezaei et al. 2019; Tsoukou et al. 2020) in continued inactivation of cells in water and microbial cells being killed by contact with water that had first been activated by discharges without being subjected to the plasma plume (Naïtali et al. 2010). The long-term, post-plasma effect is mainly caused by the reaction between H_2_O_2_ and ozone during the peroxone process that forms •OH (Magureanu et al. 2021).

The pH was 7.07 before treatment, and it went down to 3.76 after 15 min of plasma treatment (Fig. 3d). The drop in pH is similar to other studies which also achieved pH of 3.78 and 3.85, respectively, both at 15 min plasma treatment time (Pandey et al. 2023). The nitrates and nitrites led to the formation of HNO_3_, which resulted in the reduction of pH in this study. The low pH keeps the oxidizing potential of ozone at 2.08 V which can decrease to 1.4 V under alkaline conditions (Zeghioud et al. 2020). The production of hydrogen radicals also increases under acidic conditions, which then react with H_2_O_2_ and H_2_O to produce more •OH. An acid pH range of 3–4 is said to be conducive for production of •OH (Magureanu et al. 2021) and results in the increase in cell membrane permeability, enabling easy penetration of reactive molecules through the cell walls (Zhang et al. 2023).

The conductivity was 11.5 mS/cm before treatment, and it increased to 12.24 mS/cm after 15 min of plasma treatment (Fig. 3e). The ROS and RNS produced during NTP treatment result in varying conductivity of the water (Pandey et al. 2023). In one study, the conductivity fluctuated between 2.57 mS/cm and 3.31 mS/cm over 30 min of NTP treatment (Liew et al. 2023), and in another study, the conductivity increased from 1 to 123 µS/cm over 15 min treatment time (Pandey et al. 2023). Although the conductivity in this study increased with reaction time, it still remained fairly low as compared to the initial conductivity. This may be because a low conductivity favors the production of O_3_ and H_2_O_2_ which contribute to the destruction of the pollutants (Jiang et al. 2014; Zeghioud et al. 2020). The increment is said to be an indication of a loss in cell membrane integrity of bacteria (Wang et al. 2022).

### Inactivation of ARBs and ARGs

#### Evaluation of re-growth

**Fig. 4:**
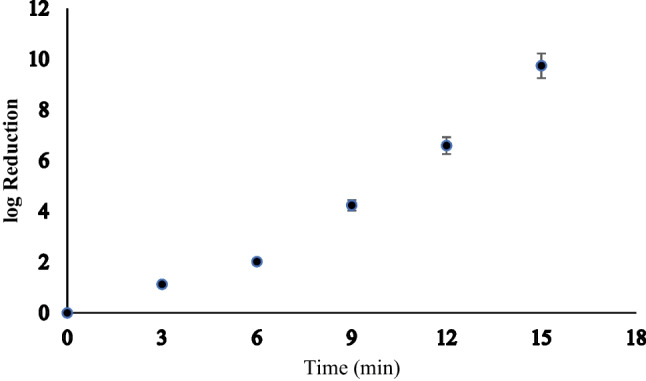
Log reduction of *A. baumannii* (BAA 1605) after non-thermal plasma treatment. Where log reduction = log_10_ (initial CFU/final CFU)

The log reductions increased with treatment time, and the highest log reduction of 9.74 ± 0.49 (close to 100% reduction) was observed after 15 min of treatment (Table 2 and Fig. 4). This indicated that NTP resulted in reduction of CRAB with time, proving that NTP be a better alternative disinfection step. Copper on its own resulted in the lowest log reduction (0.40) of *A. baumannii*, perhaps due to the solid elemental state employed. A study has shown that copper in salt form results in the greatest antimicrobial effectiveness for all the bacteria that were tested (Benhalima et al. 2019), which might be due to its ability to ionize to Cu^2+^ which makes it very toxic and reactive; hence, soluble copper catalyzes the formation of ROS, like H_2_O_2_, responsible for lipid peroxidation and DNA/RNA damage. It disrupts the binding of sulfur or iron to their respective enzymes, resulting in poor protein metallization and inactivation of the bacteria (Salah et al. 2021; Virieux-Petit et al. 2022). Moreover, it is observed that Cu^2+^ could facilitate the destruction of bacterial membrane, double-stranded DNA, and single-stranded RNA through copper-catalyzed Fenton-like reactions produced by the reactions of Cu^2+^/H_2_O_2_ (Li et al. 2021). The copper electrode used in this study did not have a significant effect on the reduction of *A. baumannii*, and it was solely the plasma discharge that resulted in the log reductions.

**Table 2:** Percentage reductions of *A. baumannii* (BAA 1605) after non-thermal plasma treatment

	Control (0 min)	Copper	3 min	6 min	9 min	12 min	15 min
CFU/mL	5.5 × 10^9^	2.2 × 10^9^	4.1 × 10^8^	5.25 × 10^7^	3.65 × 10^5^	1.4 × 10^3^	1
Log reduction	0	0.4	1.13	2.02	4.18	6.6	9.74
% reduction	0	60	92.5	99.04	99.993	≤ 100	≤ 100

NB: ≤ 100 implies that theoretical reduction was 100%; however, the presence of culturable colonies practically negates occurrence of 100% reduction

#### Evaluation of cellular disintegration

**Fig. 5:**
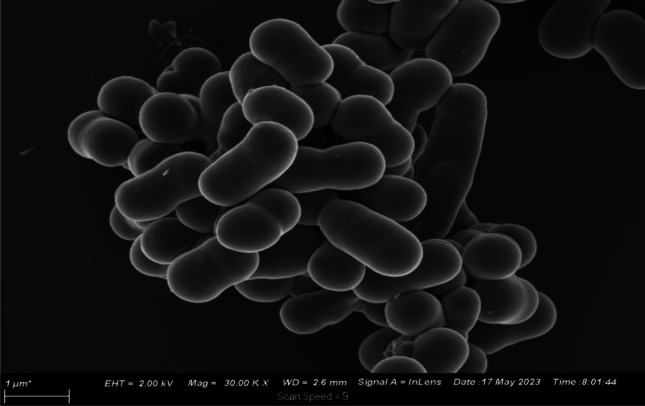
SEM image of *A. baumannii* before non-thermal plasma treatment

The cells of *A. baumannii* cells appeared smooth and coccobacilli before NTP treatment, further confirming retention of its cellular structure (Fig. 5) (Jamiu and Okesola 2023).

**Fig. 6:**
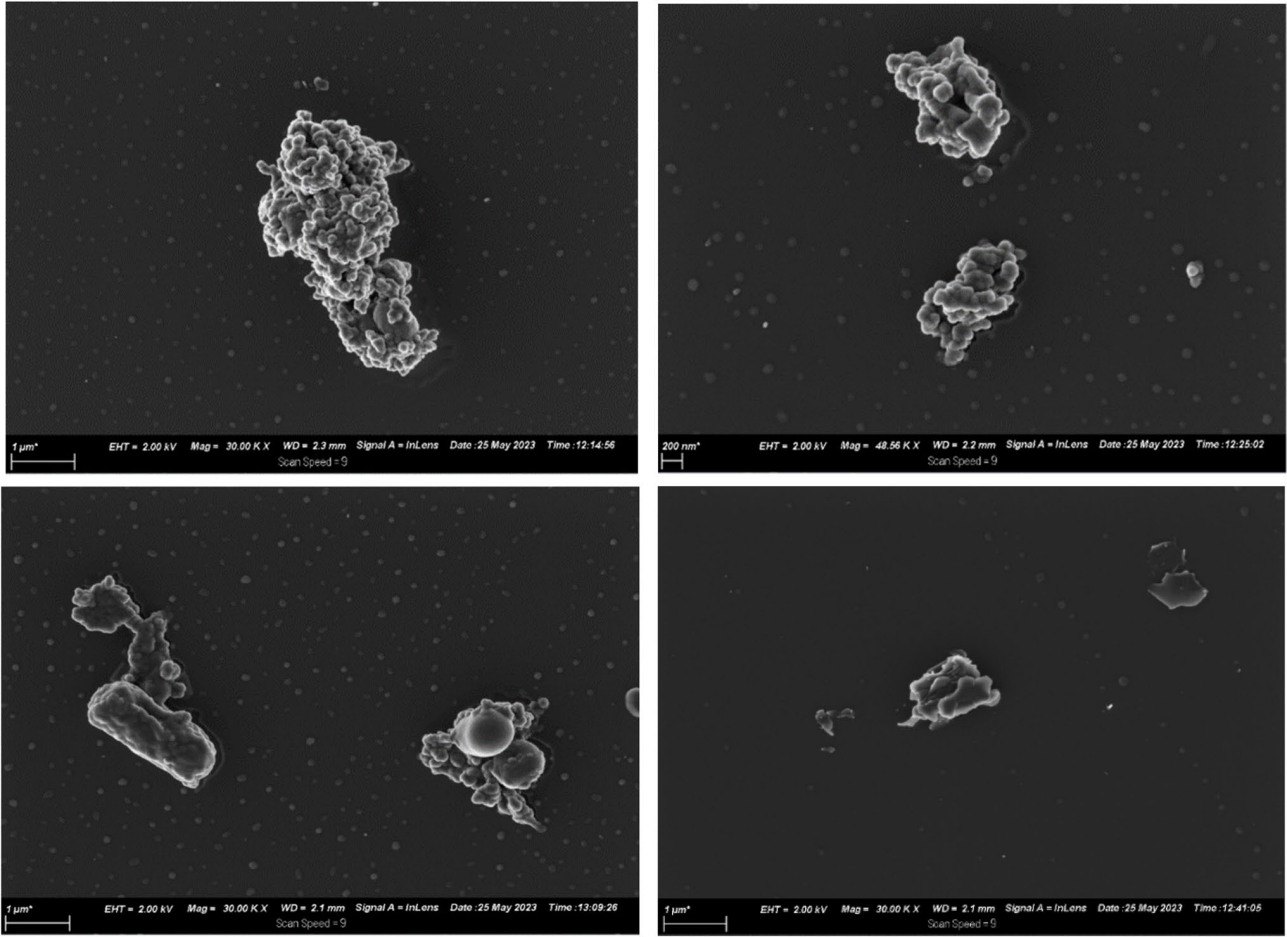
SEM images of *A. baumannii* after 15 min of non-thermal plasma treatment

However, after 15 min NTP treatment, the surface of *A. baumannii* cells had undergone significant changes (Fig. 6). The *A. baumannii* cells were destroyed and lost their characteristic coccobacilli morphology or rod shape as compared to their normal cell structure before NTP treatment. This further demonstrates that NTP interaction with the cell membrane causes it to rupture, expelling its intracellular components. Ultimately, this leads to cell death, preventing the growth of *A. baumannii* and exposing its ARGs to direct NTP attenuation (Zhang et al. 2023). This phenomenon could be attributed to the accumulation of the ROS and RNS free radicals on the cell membrane (Mazandarani et al. 2022; Zhang et al. 2023), exceeding the tensile strength of the cell membrane, rupturing the cell membrane, and eventually inactivating the bacteria (Zhang et al. 2023). Ultimately, the authors presume that the disintegration of the cellular membrane might be achieved through either or both of two phenomena: lipid peroxidation and electroporation. During lipid peroxidation, plasma-generated reactive radicals (especially OH groups) detach the polar head moieties and fatty acid tails of phosphatidyl choline residues that make up the lipid bilayer of the cell plasma membranes. This results in crosslinks between adjacent fatty acid tails, which allows unrestrained influx of radicals and water molecules, thereby causing membrane lesions and pore formation. Conversely, electric fields generated by plasma could increase transmembrane potential, which initiates lipid bilayer breakdown and membrane pore creation. The pores might be recoverable or might become irrecoverable in cases of increased electric field and time, leading to necrosis and cell rupture (Sreedevi and Suresh 2023). The fact that *A. baumannii* is a gram-negative bacterium also made it easy for NTP to destroy its membrane as it is easier for NTP to inhibit gram-negative bacteria than gram-positive bacteria (Yan et al. 2021; Zhang et al. 2023). This is because gram-negative bacteria have a thinner peptidoglycan layer and an outer membrane with components such as lipopolysaccharide (LPS) and proteins which are sensitive to ROS (Zhang et al. 2023).

**Fig. 7:**
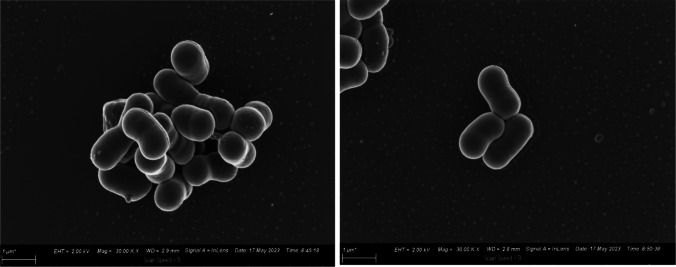
Scanning electron microscopic images of *A. baumannii* after copper treatment

Copper is capable of forming ions (Cu^+^ and Cu^2+^), which damage the membrane and infiltrate the cell and induce oxidative stress response involving endogenous ROS. However, the *A. baumannii* cells in this study were not distorted by copper treatment, maintaining their coccobacilli or rod shape throughout the treatment (Fig. 7). This might be because the copper employed in the study was not in ionic form, or because the outer membrane of gram-negative bacteria makes them less susceptible to antibacterial agents (Salah et al. 2021). A study compared SEM images of (*E. coli*) gram-negative and (*Staphylococcus aureus*) gram-positive cell after plasma treatment, the destruction was more visible on the *E. coli* cells as there was cell breakage effects on the *E. coli* cells and only shrinkage and irregular shape on the *S. aureus* cells (Han et al. 2016).

#### Detection of resistance gene (*bla*_*NDM-1*_) attenuation

**Fig. 8:**
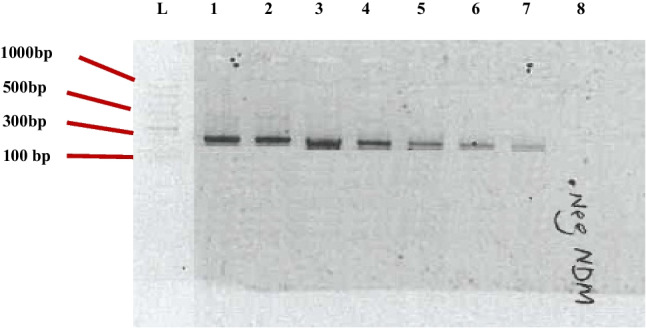
Effect of periodic non-thermal plasma treatment on the carbapenem-resistant gene of *A. baumannii*. L: DNA ladder, 1: + ve control, 2: copper control, 3: 3 min, 4: 6 min, 5: 9 min, 6: 12 min, 7: 15 min, and 8: -ve control

According to Bradford et al. (2005), the concentration of the present DNA is indicated by the fluorescence of the bands. Therefore, dull bands have a lower concentration of DNA as compared to the bands that are brighter. The band size of our *bla*_NDM-1_ was evaluated to be 230 bp, and its brightness decreased with time, illustrating that the genes were inactivated with time (Fig. 8). Although the genes were not completely inactivated after 15 min, the progression in time showed a considerable reduction in *bla*_NDM-1_, which we presume might be totally eliminated with increased time and/or electric field. Interestingly, our results are indeed remarkable, when compared with a study that employed plasma-generated Fenton-oriented reactions (Cu^2+^/H_2_O_2_ and Fe^2+^/H_2_O_2_) and recorded measurable gene copies of *bla*_TEM-1_ after 10 min, despite achieving enhanced ARG inactivation by Cu^2+^ and Fe^2+^ (Li et al. 2021). Chlorination achieved a maximum reduction of 100% (Mao et al. 2015), UV achieved a maximum reduction of 99% (Chen et al. 2020a), and ozone achieved a maximum of 98.1% (Jäger et al. 2018) of ARGs. But these came at a cost to the environment as chlorine forms harmful by-products, such as halo-organics (Luukkonen et al. 2014; Anthony et al. 2020) and ozone forms bromate (Luukkonen et al. 2014; Anthony et al. 2020), as concentrations of the disinfectants used were impractical and much higher than those currently used in WWTPs (Zhang et al. 2017; Wallmann et al. 2021; Umar 2022). The good thing about NTP is even with an increase in reaction time, no harmful chemicals are used. The grounded electrode surface or diameter can also be increased at fixed discharge gap in order to optimize the area of plasma discharge. Oxygen-containing feeding gas can also be used, instead of air and argon, and the least removal occurs in nitrogen-containing feed gas as it has been widely demonstrated to result in the fastest degradation of contaminants degradation. This is because oxygen-containing gas has been widely demonstrated to result in the fastest degradation of contaminants as it induces the production of O-based active species and O_3_ that boosts the production of •OH (Jiang et al. 2014; Magureanu et al. 2021).

## Conclusions

The 15-min treatment demonstrated the most pronounced impact on *A. baumannii* reduction, coinciding with a diminishing intensity of the resistance gene in *A. baumannii* over time. This observation, coupled with the log reductions, substantiates the temporal efficacy of NTP in completely eradicating both ARBs and ARGs. The mechanism underlying this effectiveness is attributed to the increasing presence of •OH and long-lived species (${\text{H}}_{2}{\text{O}}_{2}$, ${\text{NO}}_{2}^{-}$, and ${\text{NO}}_{3}^{-}$), which exhibit a time-dependent augmentation. These species facilitate the generation of more •OH and participate in post-discharge reactions, significantly contributing to the antibacterial activity of plasma-activated water (PAW). Concurrently, they contribute to pH reduction to levels conducive for bacterial destruction, while the expected low conductivity is maintained. Interestingly, SEM results validate the theory that CAP disrupts the tensile strength of the cell membrane, inducing rupture and consequent cell death, thereby preventing bacterial growth, including the presumed preservation of ARGs. The study excludes the antimicrobial contribution of copper, emphasizing the sole attribution of log reductions to NTP, as corroborated by SEM findings. These collective results underscore the promising potential of plasma treatment as an efficient disinfection step for wastewater. However, it is advisable to extend the treatment duration as well as increase electric field supply for ARGs, given that the observed inactivation of ARBs may not correspond to the inactivation of ARGs. This recommendation aligns with the pursuit of conclusive gene elimination outcomes.

With regard to the potential of this technology, the development of continuous flow dielectric barrier discharge and corona discharge reactors provides a great opportunity for scale-up. For example, in the work of Naicker et al. (2023), the authors investigated the potential of the non-thermal plasma in the treatment of effluent in the tertiary stage of the wastewater treatment plant and the cost with retrofitting the technology to other existing ones towards the improvement of the quality of the effluent that is discharged into the river. Moreover, the review by Mosaka et al. (2023) also highlighted the importance of the non-thermal plasma technology and the challenges that still needs to be overcome in its large-scale applicability. Overall, this technology demonstrates a great potential for the deactivation of the antibiotic-resistant bacteria and their genes in a batch reactor, while the reactor design for implementation for scale-up is being considered from the perspective of the treatment of hospital wastewater.

## Data Availability

Sources of data collected have been mentioned in the text.

## References

[CR1] Anand R, Ellappan K, Narasimha H (2015) Prevalence and characterization of NDM-1 and OXA-48 carbapenemase gene harboring Enterobacteriaceae in a tertiary care hospital, South India. Afr J Bacteriol Res 7:60–63

[CR2] Anthony ET, Ojemaye MO, Okoh OO, Okoh AI (2020) A critical review on the occurrence of resistomes in the environment and their removal from wastewater using apposite treatment technologies: limitations, successes and future improvement. Environ Pollut 263:113791 32224385 10.1016/j.envpol.2019.113791

[CR3] Azuma T, Usui M, Hayashi T (2022) Inactivation of antibiotic-resistant bacteria in wastewater by ozone-based advanced water treatment processes. Antibiotics 11:210 35203813 10.3390/antibiotics11020210 PMC8868322

[CR4] Beber de Souza J, Queiroz Valdez F, Jeranoski RF, Vidal CMDS, Cavallini GS (2015) Water and wastewater disinfection with peracetic acid and UV radiation and using advanced oxidative process PAA/UV. Int J Photoenerg 1:860845

[CR5] Benhalima L, Amri S, Bensouilah M, Ouzrout R (2019) Antibacterial effect of copper sulfate against multi-drug resistant nosocomial pathogens isolated from clinical samples. Pak J Med Sci 35:1322–1328 31489000 10.12669/pjms.35.5.336 PMC6717487

[CR6] Bradford WD, Cahoon L, Freel SR, Hoopes LLM, Eckdahl TT (2005) An inexpensive gel electrophoresis-based polymerase chain reaction method for quantifying mRNA levels. Cell Biol Education 4(2):157–168 10.1187/cbe.04-09-0051 PMC1103717 15917874

[CR7] Chen L, Xu Y, Dong X, Shen C (2020a) Removal of intracellular and extracellular antibiotic resistance genes in municipal wastewater effluent by electrocoagulation. Environ Eng Sci 37:783–789 10.1089/ees.2020.0189

[CR8] Chen L, Zhou Z, Shen C, Xu Y (2020b) Inactivation of antibiotic-resistant bacteria and antibiotic resistance genes by electrochemical oxidation/electro-Fenton process. Water Sci Technol 81:2221–2231 32701499 10.2166/wst.2020.282

[CR9] Chen Y, Duan X, Zhou X, Rupeng W, Wang S, Ren N-Q, Ho S-H (2020c) Advanced oxidation processes for water disinfection: features, mechanisms and prospects. Chem Eng J 409:128207 10.1016/j.cej.2020.128207

[CR10] Das S, Gajula VP, Mohapatra S, Singh G, Kar S (2022) Role of cold atmospheric plasma in microbial inactivation and the factors affecting its efficacy. Health Sci Rev 4:100037 10.1016/j.hsr.2022.100037

[CR11] Dekic S, Hrenovic J, Van Wilpe E, Venter C, Goic-Barisic I (2019) Survival of emerging pathogen *Acinetobacter baumannii* in water environment exposed to different oxygen conditions. Water Sci Technol 80:1581–1590 31961820 10.2166/wst.2019.408

[CR12] Domonkos M, Tichá P, Trejbal J, Demo P (2021) Applications of cold atmospheric pressure plasma technology in medicine, agriculture and food industry. Appl Sci 11:4809 10.3390/app11114809

[CR13] Ebomah KE, Okoh AI (2020) An African perspective on the prevalence, fate and effects of carbapenem resistance genes in hospital effluents and wastewater treatment plant (WWTP) final effluents: a critical review. Heliyon 6:e03899 32420480 10.1016/j.heliyon.2020.e03899 PMC7215200

[CR14] Ekwanzala MD, Dewar JB, Kamika I, Momba MNB (2018) Systematic review in South Africa reveals antibiotic resistance genes shared between clinical and environmental settings. Infect Drug Resist 11:1907–1920 30425540 10.2147/IDR.S170715 PMC6203169

[CR15] Fishbain J, Peleg AY (2010) Treatment of Acinetobacter infections. Clin Infect Dis 51:79–84 20504234 10.1086/653120

[CR16] Foster J (2017) Plasma-based water purification: challenges and prospects for the future. Phys Plasmas 24:055501 10.1063/1.4977921

[CR17] Gmbh T (2021) SpectroDirect / PC Spectro II_8c 02/2021 [Online]. Germany: Tintometer GmbH. Available: https://www.lovibond.com/ix_pim_assets/Wasseranalytik/Instruction_Manuals/Photometer/SpectroDirect/ins_spectrodirect_gb_lovi.pdf. Accessed 15 January 2024

[CR18] Gwanzura E, Awolusi OO, Kumari S, Ramjugernath D, Iwarere SA (2021) An electrohydraulic direct current discharge for inactivation of *Escherichia coli* in high-bacterial density wastewaters. Int J Eng Res Afr 55:190–206 10.4028/www.scientific.net/JERA.55.190

[CR19] Han L, Patil S, Boehm D, Milosavljević V, Cullen PJ, Bourke P (2016) Mechanisms of inactivation by high-voltage atmospheric cold plasma differ for Escherichia coli and Staphylococcus aureus. Appl Environ Microbiol 82:450–458 26519396 10.1128/AEM.02660-15 PMC4711144

[CR20] Howard A, O’donoghue M, Feeney A, Sleator RD (2012) Acinetobacter baumannii: an emerging opportunistic pathogen. Virulence 3:243–50 22546906 10.4161/viru.19700 PMC3442836

[CR21] Hrenović J, Goic-Barišić I, Kazazić S, Kovačić A, Ganjto M, Tonkic M (2016) Carbapenem-resistant isolates of Acinetobacter baumannii in a municipal wastewater treatment plant, Croatia, 2014. Eurosurveillance 21(15):30195 10.2807/1560-7917.ES.2016.21.15.30195 10.2807/1560-7917.ES.2016.21.15.30195 27105318

[CR22] Jäger T, Hembach N, Elpers C, Wieland A, Alexander J, Hiller C, Krauter G, Schwartz T (2018) Reduction of antibiotic resistant bacteria during conventional and advanced wastewater treatment, and the disseminated loads released to the environment. Front Microbiol 9:2599 30425704 10.3389/fmicb.2018.02599 PMC6218952

[CR23] Jamiu MO, Okesola A (2023) *Acinetobacter baumannii*: an emerging threat to public health - a review of literature. Int J Curr Sci Res Rev 6:1848–1864

[CR24] Jiang B, Zheng J, Qiu S, Wu M, Zhang Q, Yan Z, Xue Q (2014) Review on electrical discharge plasma technology for wastewater remediation. Chem Eng J 236:348–368 10.1016/j.cej.2013.09.090

[CR25] Jin M, Liu L, Wang DN, Yang D, Liu WL, Yin J, Yang ZW, Wang HR, Qiu ZG, Shen ZQ, Shi DY, Li HB, Guo JH, Li JW (2020) Chlorine disinfection promotes the exchange of antibiotic resistance genes across bacterial genera by natural transformation. Isme J 14:1847–1856 32327733 10.1038/s41396-020-0656-9 PMC7305130

[CR26] Lee PY, Costumbrado J, Hsu CY, Kim YH (2012) Agarose gel electrophoresis for the separation of DNA fragments. J vis Exp 62:e3923 10.3791/3923 PMC4846332 22546956

[CR27] Li H, Song R, Wang Y, Zhong R, Zhang Y, Zhou J, Wang T, Jia H, Zhu L (2021) Inhibited conjugative transfer of antibiotic resistance genes in antibiotic resistant bacteria by surface plasma. Water Res 204:117630 34536683 10.1016/j.watres.2021.117630

[CR28] Liew KJ, Zhang X, Cai X, Ren D, Chen J, Chang Z, Chong K, Tan Chun Yun M, Chong CS (2023) The biological responses of Staphylococcus aureus to cold plasma treatment. Processes 11(4):1188 10.3390/pr11041188

[CR29] Luukkonen T, Teeriniemi J, Prokkola H, Rämö J, Lassi U (2014) Chemical aspects of peracetic acid based wastewater disinfection. Water SA 40:73–80 10.4314/wsa.v40i1.9

[CR30] Magureanu M, Bilea F, Bradu C, Hong D (2021) A review on non-thermal plasma treatment of water contaminated with antibiotics. J Hazard Mater 417:125481 33992019 10.1016/j.jhazmat.2021.125481

[CR31] Manaia CM, Rocha J, Scaccia N, Marano R, Radu E, Biancullo F, Cerqueira F, Fortunato G, Iakovides IC, Zammit I, Kampouris I, Vaz-Moreira I, Nunes OC (2018) Antibiotic resistance in wastewater treatment plants: tackling the black box. Environ Int 115:312–324 29626693 10.1016/j.envint.2018.03.044

[CR32] Mao D, Yu S, Rysz M, Luo Y, Yang F, Li F, Hou J, Mu Q, Alvarez PJ (2015) Prevalence and proliferation of antibiotic resistance genes in two municipal wastewater treatment plants. Water Res 85:458–466 26372743 10.1016/j.watres.2015.09.010

[CR33] Mazandarani A, Goudarzi S, Jafarabadi M, Azimi Nekoo E (2022) Effects of cold plasma on *Staphylococcus Aureus*. J Fam Reprod Health 16:212–216 10.18502/jfrh.v16i3.10583 PMC9759435 36569254

[CR34] Mosaka TB, Unuofin JO, Daramola MO, Tizaoui C. Iwarere SA (2023) Inactivation of antibiotic-resistant bacteria and antibiotic-resistance genes in wastewater streams: Current challenges and future perspectives. Front Microbiol 13:1100102 10.3389/fmicb.2022.1100102 PMC9888414 36733776

[CR35] Naicker K-I, Kaweesa P, Daramola MO, Iwarere SA (2023) Non-thermal plasma review: assessment and improvement of feasibility as a retrofitted technology in tertiary wastewater purification. Appl Sci 13:6243 10.3390/app13106243

[CR36] Naïtali M, Kamgang-Youbi G, Herry JM, Bellon-Fontaine MN, Brisset JL (2010) Combined effects of long-living chemical species during microbial inactivation using atmospheric plasma-treated water. Appl Environ Microbiol 76:7662–7664 20889799 10.1128/AEM.01615-10 PMC2976197

[CR37] Nijdam S, Teunissen J, Ebert U (2020) The physics of streamer discharge phenomena. Plasma Sources Sci Technol 29(10):103001 10.1088/1361-6595/abaa05

[CR38] Odjadjare EC, Olaniran AO (2015) Prevalence of antimicrobial resistant and virulent Salmonella spp. in treated effluent and receiving aquatic milieu of wastewater treatment plants in Durban, South Africa. Int J Environ Res Public Health 12:9692–9713 26295245 10.3390/ijerph120809692 PMC4555307

[CR39] Ortega-Nieto C, Losada-Garcia N, Pessela B, Domingo-Calap P, Palomo JM (2023) Design and synthesis of copper nanobiomaterials with antimicrobial properties. ACS Bio Med Chem Au 3(4):349–358 10.1021/acsbiomedchemau.2c00089 PMC10436259 37599792

[CR40] Pandey S, Jangra R, Ahlawat K, Mishra R, Mishra A, Jangra S, Prakash R (2023) Selective generation of nitrate and nitrite in plasma activated water and its physicochemical parameters analysis. Phys Lett A 474:128832 10.1016/j.physleta.2023.128832

[CR41] Picetti R, Deeney M, Pastorino S, Miller MR, Shah A, Leon DA, Dangour AD, Green R (2022) Nitrate and nitrite contamination in drinking water and cancer risk: a systematic review with meta-analysis. Environ Res 210:112988 35217009 10.1016/j.envres.2022.112988

[CR42] Pulami D, Kämpfer P, Glaeser SP (2023) High diversity of the emerging pathogen Acinetobacter baumannii and other Acinetobacter spp. in raw manure, biogas plants digestates, and rural and urban wastewater treatment plants with system specific antimicrobial resistance profiles. Sci Total Environ 859:160182 36395844 10.1016/j.scitotenv.2022.160182

[CR43] Querci M, Kagkli D, Gatto F, Foti N, Maretti M, Mazzara M (2020) The analysis of food samples for the presence of Genetically Modified Organisms - User Manual, EUR 30145 EN, Publications Office of the European Union, Luxembourg. 10.2760/5277

[CR44] Rashmei Z, Bornasi H, Ghoranneviss M (2016) Evaluation of treatment and disinfection of water using cold atmospheric plasma. J Water Health 14:609–616 27441856 10.2166/wh.2016.216

[CR45] Raut S, Rijal KR, Khatiwada S, Karna S, Khanal R, Adhikari J, Adhikari B (2020) Trend and characteristics of Acinetobacter baumannii infections in patients attending Universal College of Medical Sciences, Bhairahawa, Western Nepal: a longitudinal study of 2018. Infect Drug Resist 13:1631–1641 32606814 10.2147/IDR.S257851 PMC7293404

[CR46] Reinke RA, Quach-Cu J, Allison N, Lynch B, Crisostomo C, Padilla M (2020) A method to quantify viable carbapenem resistant gram-negative bacteria in treated and untreated wastewater. J Microbiol Methods 179:106070 33017624 10.1016/j.mimet.2020.106070

[CR47] Rekhate CV, Srivastava JK (2020) Recent advances in ozone-based advanced oxidation processes for treatment of wastewater- a review. Chem Eng J Adv 3:100031 10.1016/j.ceja.2020.100031

[CR48] Rezaei F, Vanraes P, Nikiforov A, Morent R, De Geyter N (2019) Applications of plasma-liquid systems: a review. Materials 12:2751 31461960 10.3390/ma12172751 PMC6747786

[CR49] Salah I, Parkin I, Allan E (2021) Copper as an antimicrobial agent: recent advances. RSC Adv 11:18179–18186 35480904 10.1039/D1RA02149D PMC9033467

[CR50] Sanito RC, You S-J, Wang Y-F (2022) Degradation of contaminants in plasma technology: an overview. J Hazard Mater 424:127390–127390 34879580 10.1016/j.jhazmat.2021.127390 PMC8500698

[CR51] Sarangapani C, Ziuzina D, Behan P, Boehm D, Gilmore B, Cullen PJ, Bourke P (2019) Degradation kinetics of cold plasma-treated antibiotics and their antimicrobial activity. Sci Rep 9:3955 30850645 10.1038/s41598-019-40352-9 PMC6408491

[CR52] Sharma VK, Yu X, Mcdonald TJ, Jinadatha C, Dionysiou DD, Feng M (2019) Elimination of antibiotic resistance genes and control of horizontal transfer risk by UV-based treatment of drinking water: a mini review. Front Environ Sci Eng 13:1–9 10.1007/s11783-019-1122-7 PMC7055709 32133212

[CR53] Shi J, Sun T, Cui Y, Wang C, Wang F, Zhou Y, Miao H, Shan Y, Zhang Y (2020) Multidrug resistant and extensively drug resistant *Acinetobacter baumannii* hospital infection associated with high mortality: a retrospective study in the pediatric intensive care unit. BMC Infect Dis 20:597 32787942 10.1186/s12879-020-05321-y PMC7422664

[CR54] Soni K, Jyoti K, Chandra H, Chandra R (2022) Bacterial antibiotic resistance in municipal wastewater treatment plant; mechanism and its impacts on human health and economy. Bioresour Technol Rep 19:101080 10.1016/j.biteb.2022.101080

[CR55] Sreedevi PR, Suresh K (2023) Cold atmospheric plasma mediated cell membrane permeation and gene delivery-empirical interventions and pertinence. Adv Coll Interface Sci 320:102989 10.1016/j.cis.2023.102989 37677997

[CR56] Tsoukou E, Bourke P, Boehm D (2020) Temperature stability and effectiveness of plasma-activated liquids over an 18 months period. Water 12(11):3021. 10.3390/w12113021 10.3390/w12113021

[CR57] Umar M (2022) From conventional disinfection to antibiotic resistance control-status of the use of chlorine and UV irradiation during wastewater treatment. Int J Environ Res Public Health 19(3):1636. 10.3390/ijerph19031636 35162659 10.3390/ijerph19031636 PMC8834887

[CR58] Unuofin JO (2020) Garbage in garbage out: the contribution of our industrial advancement to wastewater degeneration. Environ Sci Pollut Res 27:22319–22335 10.1007/s11356-020-08944-5 32347482

[CR59] Valencia-Martín R, Gonzalez-Galan V, Alvarez-Marín R, Cazalla-Foncueva AM, Aldabó T, Gil-Navarro MV, Alonso-Araujo I, Martin C, Gordon R, García-Nuñez EJ, Perez R, Peñalva G, Aznar J, Conde M, Cisneros JM et al (2019) A multimodal intervention program to control a long-term Acinetobacter baumannii endemic in a tertiary care hospital. Antimicrob Resist Infect Control 8:199 31827780 10.1186/s13756-019-0658-4 PMC6894224

[CR60] Viehman JA, Nguyen MH, Doi Y (2014) Treatment options for carbapenem-resistant and extensively drug-resistant *Acinetobacter baumannii* infections. Drugs 74:1315–33 25091170 10.1007/s40265-014-0267-8 PMC4258832

[CR61] Virieux-Petit M, Hammer-Dedet F, Aujoulat F, Jumas-Bilak E, Romano-Bertrand S (2022) From copper tolerance to resistance in Pseudomonas aeruginosa towards patho-adaptation and hospital success. Genes 13:301 35205346 10.3390/genes13020301 PMC8872213

[CR62] Wallmann L, Krampe J, Lahnsteiner J, Radu E, Van Rensburg P, Slipko K, Wögerbauer M, Kreuzinger N (2021) Fate and persistence of antibiotic-resistant bacteria and genes through a multi-barrier treatment facility for direct potable reuse. J Water Reuse Desalination 11:373–390 10.2166/wrd.2021.097

[CR63] Wang Y, Han Y, Li L, Liu J, Yan X (2022) Distribution, sources, and potential risks of antibiotic resistance genes in wastewater treatment plant: a review. Environ Pollut 310:119870 35921944 10.1016/j.envpol.2022.119870

[CR64] World Health Organisation (2024) WHO Bacterial Priority Pathogens List, 2024: bacterial pathogens of public health importance to guide research, development and strategies to prevent and control antimicrobial resistance. Geneva: World Health Organization; 2024 [Online]. Available: https://www.who.int/publications/i/item/9789240093461. Accessed 23 June 2024

[CR65] Yan D, Malyavko A, Wang Q, Ostrikov KK, Sherman JH, Keidar M (2021) Multi-modal biological destruction by cold atmospheric plasma: capability and mechanism. Biomedicines 9(9):1259. 10.3390/biomedicines9091259 34572443 10.3390/biomedicines9091259 PMC8465976

[CR66] Yuan Q-B, Guo M-T, Yang J (2015) Fate of antibiotic resistant bacteria and genes during wastewater chlorination: implication for antibiotic resistance control. PLoS One 10:e0119403 25738838 10.1371/journal.pone.0119403 PMC4349789

[CR67] Zeghioud H, Nguyen-Tri P, Khezami L, Amrane A, Assadi AA (2020) Review on discharge plasma for water treatment: mechanism, reactor geometries, active species and combined processes. J Water Process Eng 38:101664 10.1016/j.jwpe.2020.101664

[CR68] Zhang CM, Xu LM, Wang XC, Zhuang K, Liu QQ (2017) Effects of ultraviolet disinfection on antibiotic-resistant Escherichia coli from wastewater: inactivation, antibiotic resistance profiles and antibiotic resistance genes. J Appl Microbiol 123:295–306 28459506 10.1111/jam.13480

[CR69] Zhang T, Zhou R, Wang P, Mai-Prochnow A, Mcconchie R, Li W, Zhou R, Thompson EW, Ostrikov K, Cullen PJ (2021) Degradation of cefixime antibiotic in water by atmospheric plasma bubbles: performance, degradation pathways and toxicity evaluation. Chem Eng J 421:127730 10.1016/j.cej.2020.127730

[CR70] Zhang H, Zhang C, Han Q (2023) Mechanisms of bacterial inhibition and tolerance around cold atmospheric plasma. Appl Microbiol Biotechnol 107:5301–5316 37421472 10.1007/s00253-023-12618-w PMC10390405

